# Metabolic Syndrome and the Immunological Affair with the Blood–Brain Barrier

**DOI:** 10.3389/fimmu.2014.00677

**Published:** 2015-01-05

**Authors:** Claudio Mauro, Veronica De Rosa, Federica Marelli-Berg, Egle Solito

**Affiliations:** ^1^William Harvey Research Institute, Barts and The London School of Medicine and Dentistry, Queen Mary University of London, London, UK; ^2^Istituto Per L’Endocrinologia e L’Oncologia Sperimentale ”G.Salvatore” – Consiglio Nazionale delle Ricerche (IEOS-CNR), Naples, Italy

**Keywords:** blood–brain barrier, metabolism, immune response, leukocytes, diet

## Abstract

Epidemiological studies reveal an increased incidence of obesity worldwide, which is associated with increased prevalence and severity of cognitive disorders. The blood–brain barrier (BBB) represents the interface between the peripheral circulation and the brain, and plays a fundamental role in the cross-talk between these two compartments. The homeostatic function of the BBB is the protection of the brain from peripheral insult/inflammation. Alterations in the function of the BBB lead to pathologies of the central nervous system. Recently, metabolic imbalance has been shown to be an important risk factor associated with the decline of BBB integrity and function. This has direct etiological consequences on a variety of cerebrovascular and neurodegenerative pathologies with great impact to society. Priority areas for future preclinical research include strategies to improve clinicians’ ability to diagnose, prevent, and manage BBB abnormalities. In sharp contrast with epidemiological studies and clinical needs, little is known about the mechanisms that link metabolic syndrome to BBB functionality and cognitive disorders. Our view is that immune responses caused by metabolic stress might play a major role in this conundrum.

## Introduction

Central nervous system (CNS) homeostasis is a prerequisite for the proper communication and function of neuronal cells. The endothelial blood–brain barrier (BBB) and the epithelial blood–cerebrospinal fluid barrier (BCSFB) tightly seal off the CNS from the continuously changing milieu within the bloodstream (1) by globally controlling immune cell trafficking. The BBB is a complex three-dimensional structure composed of specialized endothelial cells that are reinforced by pericytes, astrocytes end feet, and extracellular matrix (2). The extreme tightness of the BBB is due to specialized endothelial junctional complexes: the adherens junctions (AJs) and the tight junctions (TJs). TJs formation between endothelial cells is regulated by astrocytes, astrocyte-derived soluble factors as well as metalloproteases (glia limitans) and pericytes (so called neurovascular unit-NVU), which are important modulators of the BBB permeability (3). Endothelial cells also express transmembrane molecules called integrins, which are heteromeric molecules involved in anchoring the cellular structure to the sub-endothelial basal lamina/extracellular matrix (4). Integrins interact with so called “focal adhesion” molecules, which are tightly connected to the actin cytoskeleton and together with TJs and AJs are key in regulating paracellular permeability (5). We have recently reported that another important component for barrier tightness is the anti-inflammatory protein Annexin A1 (ANXA1), which has a dual role in stabilizing actin and TJ formation, and protecting the brain by promoting resolution of inflammation (6), a molecule that we propose to have great potential in correcting BBB leakage.

Since BBB damage appears to be present in many neurological disorders, it is not surprising to see much effort focused on the development of therapies targeting the barrier (7). As BBB dysfunction can either be a causative phenomenon or a propagative/exacerbating event in the course of diseases, limiting its impairment could potentially reduce the severity of pathology and facilitate recovery (5).

Equally, inflammatory mediators are the primary cause of the negative effects at the barrier level, and most of the attempts have been focused at halting the inflammatory reaction. In particular glucocorticoids like dexamethasone have shown improvement of the physical and transport properties of the BBB (8), but their usefulness, for instance, in patients suffering from multiple sclerosis (MS) decreases with time (9).

## The Immunology of BBB

The immune-surveillance of the CNS is essential to keep under control the entry of potential mediators of infection into the brain parenchyma (10). Resident microglia are the primary guardians of the brain parenchyma; however, a small number of T and B cells, macrophages, and neutrophils from the periphery lie within close proximity, patrolling the specialized CNS compartments (11). Lymphocyte recruitment across the BBB into CNS, although very low in healthy individuals, is responsible to maintain CNS immune-surveillance. Immune cells gain access to the CNS via three routes: (a) non-fenestrated vascularized stroma of the blood–cerebrospinal fluid (CSF) barrier that is surrounded by the epithelial cells of the choroid plexus, (b) the perivascular space, where the deep arteries are continuous with the subarachnoid space, and (c) post-capillary venules that enter the parenchyma directly. Extravasation migration across the vascular wall and the glial limitans propagates entry into the brain parenchyma. Within CSF of healthy individuals, the majority of immune cells (~80%) include T cells predominantly activated central memory T cells (CD4+/CD45RA−/CD27/CD69+) (12). They traffic within the CSF until encountering an antigen-presenting cell (APC) (13). In the experimental autoimmune encephalomyelitis (EAE), effector T cells entering the CNS become activated after short contacts with leptomeningeal phagocytes at the onset of disease. During established disease, the activation process is extended to the depth of the CNS parenchyma, where cells form contacts with microglia and recruited phagocytes, suggesting that they become able to infiltrate the brain parenchyma (14). Upon activation, T cells upregulate integrins and adhesion molecules, which enable leukocyte rolling and arrest at the vessel wall. Multiple integrins, cytokines, and adhesion molecules expressed on circulating and CNS-resident cells are responsible for the initial events of the immune cascade (12), which leads to leukocytes extravasation in the brain parenchyma. It has recently been shown that intravenously transferred effector T cells gain the capacity to enter the CNS after residing transiently within the lung tissues. Inside the lung tissues, they move along the airways to bronchus-associated lymphoid tissues and lung-draining mediastinal lymph nodes before entering the blood circulation from where they reach the CNS (15). On their way, T cells reprogram their gene-expression profile, characterized by downregulation of their activation status and upregulation of cellular locomotion molecules together with chemokine and adhesion receptors. The adhesion receptors include ninjurin (16), which participates in T cell intravascular crawling on cerebral blood vessels. In addition, alternative routes can be used by T cells to gain access to the CNS. In (EAE) Th17 pathogenic T cells enter the CNS via the choroid plexus, a route controlled by the CCR6–CCL20 axis (17). Furthermore, an elegant study by Arima et al. (18) clearly shows that CD4+ pathogenic T cell access the CNS trough lumbar spinal blood vessel cord being regulated by CCL20. Such data are very important in the context of neurological disease with a clear immune component.

## Metabolic Stress and BBB Disruption

It is nowadays clear that metabolic syndrome (MetS), a cluster of risk factors for cardiovascular disease, diabetes, and stroke, is becoming endemic. Epidemiological studies show that lifestyle (absence of physical exercise) and misbalanced diet (based on the so-called “junk food”) are major causes of MetS. Obesity-related disorders have risen by nearly 90% in the last decade (19). It is therefore of paramount importance to educate young and elderly people to a proper lifestyle. However, since socio-economical pressure accounts for the difficulty in meeting these targets, at the moment, early diagnosis and treatment are possible venues to halt and prevent the escalation of MetS. For instance, type 2 diabetes and Alzheimer disease (AD) have been recently pinpointed as likely linked to aging (20), clearly indicating a possible path for the identification of drugs able to control both pathologies (21).

**Figure 1:**
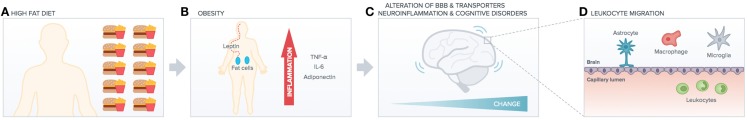
**High-fat diet impact on peripheral versus central function**. High-fat diet **(A)** induces obesity **(B)** increasing peripheral chronic inflammation (cytokines release). This has deleterious effects on brain functionality (e.g., alteration of BBB transporters, neuroinflammation, and cognitive disorders such as AD). Peripheral inflammation and increased BBB leakage **(C)** induce leukocytes migration into the brain **(D)**, which exacerbates neuroinflammation and neurodegenerative diseases.

Despite this emerging importance of the environment in triggering adaptive immunity of the CNS, a potential role for metabolic stress, an important risk factor for pro-inflammatory immune imbalance (22) and cognitive imbalance, has not been investigated so far (Figure 1). For instance, the link between metabolic stress and BBB functions is far from clear, aging factors may account for alteration in tightness, and chronic peripheral inflammation may also be accountable but no clear studies yet indicate the molecular and cellular mechanisms underlying such link. One molecule that we know is downregulated with aging is Annexin A1 (23), but no studies have related yet such molecule to diet or even metabolic syndrome. Very recently, the commensal gut flora has been shown to be essential in triggering immune responses in the brain (24), implying that the gut-associated-lymphoid-tissue (GALT) is a potential site for priming brain-targeted immune responses.

## Diet and Cerebrovascular Function

The escalating incidence of obesity worldwide has mounted large interest in studying the pathological consequences of increased fat and cholesterol intake (25) that heavily contribute to the progression of MetS. MetS develops as a consequence of low-grade chronic inflammation due to high-fat consumption and increases the risk of deleterious outcomes such as cognitive impairment (25), stroke (26), and neurodegenerative conditions such as Alzheimer’s disease (AD) (27) and MS (28).

Although the BBB is exposed to both the peripheral and central environments, the effects of obesity and MetS are bleak. In addition, age and excessive energy intake/obesity are reported as risk factors for cerebrovascular disease. A recent study suggested that reduction in neurofactors (named brain-derived neurotrophic factor and basic fibroblast growth factor) as well as inflammatory pathways (the antioxidant enzyme heme oxygenase-1) with aging is responsible for the poor outcome post-infarction in aging (26). Moreover, epidemiological studies on the effect of western diet on learning and memory are ascribed to BBB degradation (29).

## Cerebral Metabolism and BBB Transporters

Being one of the most metabolically active organs in the body, the brain does not store excess energy and derives almost all of its energetic needs from the aerobic oxidation of glucose. Therefore, it requires continuous supply of glucose and oxygen to meet its energy demands. Glucose can enter the brain from the blood through two different routes: (a) the sodium-dependent glucose transporters (SGLTs) and (b) the sodium-independent glucose transporters (GLUTs). GLUT-1 is expressed in brain capillary endothelial cells (30, 31) and safeguards glucose transport across the BBB. Glucose transport and transporters are also affected by systemic autoimmune-inflammation, for example, GLUT-1 is up-regulated by interleukin-1β (IL-1β) at endothelial level although its effects at neuronal level are deleterious (32). Therefore, one could argue that since metabolism is associated with a degree of on-going inflammation, the alteration of glucose transport and transporters at the BBB could impact on brain networking resulting in learning deficit in young obese individuals and in cognitive impairment in aged subjects. Moreover, another important BBB transporter member of the ATP-binding cassette protein family, the P-glycoprotein (which transports various molecules in and out the brain parenchyma) appears to be modulated both in its activity and expression by inflammatory events occurring at the level of the BBB endothelium (33). Consequently, as P-glycoprotein activity is important for the passage of therapeutic molecules through the BBB, understanding its regulation during inflammation would aid in the development of drugs (32, 33).

Another important molecule with different functions in the CNS compared to the peripheral system is insulin. Brain endothelial cells (BEC) contain saturable transporter pores (34) that translocate insulin from the blood to the brain. Produced most exclusively by the pancreas, insulin crosses the BBB affecting feeding and cognition (35). Similar to insulin, leptin (secreted by adipocytes) crosses the BBB through a saturable transport mechanism possibly independent of insulin (36) regulating appetite and energy balance (37). Moreover, leptin produced by adipocytes and lymphocytes (metabolic inflammation) has pro-inflammatory activity possibly contributing to CNS inflammation (38).

## BBB Function and Pathology

The composition and structural organization of the BBB is key to maintaining a constant and optimal cerebral environment for neuronal function through a combination of barriers and selective transport systems that tightly regulate the passage of essential and unwanted molecules (39). Nowadays, the BBB is not considered as a static, passive structure that serves as an impediment to molecular access into the CNS (40). In contrast, it modulates and actively filters molecules and blood born cells into the CNS, and functions as a highly specialized transport, metabolic, physical, and immunological barrier. Consequently, alterations in BBB integrity and function are associated with many pathologies of the CNS (41), namely, MS, hypoxia and ischemia, edema, Parkinson’s disease (PD) and AD, epilepsy, glaucoma, and lysosomal storage diseases (3). Barrier dysfunction can range from mild and transient TJ opening to chronic barrier breakdown and changes in transport systems and enzymes (5). Breakdown of the BBB allows immune cell infiltration to aid clearing debris and repair injuries. However, in several cases, it results in damage to the CNS, causing neuronal dysfunction, injury, and degeneration (41). Hence, it is not clear if changes in BBB physiology should be considered as one of the causes of the disease, part of the pathophysiological process, a consequence of the disease, or a combination of all. Nonetheless, recent evidence supports the hypothesis of endothelial dysfunction as a link between vascular disorders and neurological impairment, exacerbating the development of CNS disorders (40, 42).

## Metabolic Imbalance and Cognitive Decline

A link between nutrition and mental health has been recently established. Many eating-related peptides and regulatory proteins produced by peripheral tissues and with receptors in the brain have been found to cross the BBB. Thus, the fact that BBB results permeable to factors that are important for the brain functionality can be seen under the umbrella of BBB physiological conduits toward the control of signaling between the peripheral and central system. Consequently, dysfunction of BBB and its transporters can result in disease. Resistance to leptin caused by its decreased transport across the BBB in obesity is an example (37). Although rare, patients with Glut-1 deficiency (caused by genetic mutations) can have severe learning difficulties that may be corrected through the diet (43).

Impairment of insulin transport due to change of BBB component such as pericytes is also at the origin of pathological manifestation in diabetes and hyperglycemia. Resistance to insulin may occur also in the brain (so called diabetes mellitus type III) and it may or not be linked to peripheral resistance, as for ADs (35).

However, additional consideration has been put forward suggesting that prolonged increased high-fat intake may be linked to inflammatory and aging-related neurodegenerative diseases including MS, PD, and AD (44). The development of various neurodegenerative disorders have been associated with BBB damage and increased permeability. Thus, it is thought that obesity can potentially activate the onset of vascular disorders that affect BBB permeability later in life (29, 45).

Moreover, BBB modifications are often characterized by the disturbance of endothelial glial interactions (2). In addition, decreased number of TJ proteins, in particular Claudin-5 and Claudin-12, is found within the BBB and choroid plexus, increasing the permeability of the barriers and enabling the entry of toxic molecules (29, 46). During BBB disruption, agrin (a large proteoglycan) is lost from the abluminal surface of the BEC. This is thought to contribute to BBB damage in AD. Amyloid-B accumulation, a key characteristic of AD is first seen in the neighboring hood of blood vessels with toxicity on endothelium and astrocytes, before causing extensive neuronal loss and CNS homeostatic imbalance (47).

Population studies are now more and more aligned to the concept that MetS has a negative impact on learning and cognition. Multiple factors and etiology spanning from impaired vascular reactivity to autoimmune-inflammation and oxidative stress are potential factors affecting brain functionality as exhaustively reported in Ref. (48). Insulin resistance and diabetes are indeed strongly associated with deficiency in cerebrovascular functionality. Indeed, it has recently been reported that insulin-signaling dysfunction in AD may be treated with administration of intranasal insulin, which has been reported to improve mood and behavior in diabetic patients (21). Such treatment has great potential because of the beneficial effects not only on the functionality of peripheral organs but also of the brain. Metformin together with amylin and leptin analogs also deserve better investigation of their potential beneficial effects in CNS pathologies that arise as consequence of metabolic syndrome (21).

## Metabolism and Adaptive Immunity in the Peripheral–Central Axis

Although different metabolic needs and activity certainly follow changes in signaling and proliferation rate, recent evidence suggests that the regulation of T cell metabolism is tightly linked to T cell function and differentiation. T lymphocytes are finely regulated by signals that, once delivered through T cell receptor (TCR) and cytokine receptors, induce the activation of different intracellular metabolic pathways (49). Signals deriving from growth factors and cytokines such as IL-2 or IL-7, together with ligation of co-stimulatory molecules, lead to an increase in glucose uptake and glycolysis through induction of phosphoinositide-3-kinase (PI3K)-dependent activation of Akt (50, 51). T cell activation is also accompanied by an increased rate of protein synthesis, which supports cell growth and effector functions. Downstream of TCR and CD28, Akt controls the activation of the mammalian Target of Rapamycin, mTOR, which is the main regulator of protein synthesis in T cells (52, 53). The importance of the mTOR pathway for T cell activation is testified by the evidence that rapamycin, a selective inhibitor of mTOR, induces a condition of immunosuppression, through the induction of a cell cycle arrest in proliferating T lymphocytes. It has recently been shown that changes in nutritional status of the host can directly affect survival and proliferation of pro-inflammatory CD4+ T cells. Of note, dietary restriction causes metabolic and physiological changes that have beneficial effects in different pathological conditions, such as obesity, insulin resistance, inflammation, oxidative stress, and autoimmune diseases (54–56). The overall increase of obesity has focused the attention on the biology of adipose tissue, so far considered only a “passive” energy storage site. It is well accepted now that the adipose tissue can also produce hormones and cytokines, named “adipokines,” that bridge metabolism and immune homeostasis, such as leptin, IL-1β, IL-6, IL-8, interferon-γ (IFN-γ), tumor necrosis factor-α (TNF-α), transforming growth factor-β (TGF-β), leukemia-inhibiting factor (LIF), and many chemokines (57, 58). Leptin is produced by the adipose tissue in proportion to the body fat mass; its role is to regulate body weight through the inhibition of food intake and stimulation of energy expenditure but evidence has indicated that leptin is also one of the main regulators converging signals from the environment (food availability) to effector (Teff)/regulatory (Treg) T cell proliferation both *in vitro* and *in vivo* (59, 60). The evidence that adipose tissue has an important role on the control of central functions, such as immunity and metabolism, is providing novel insights into the pathogenesis of metabolic and inflammatory disorders. Leptin secreted by adipocytes sustains Th1 responses by promoting Teff cell proliferation and pro-inflammatory cytokine production and by constraining Treg cells expansion: this balance between Teff and Treg cells leads to immune tolerance on one side and to protection from infections on the other (59, 60). Recent evidence suggests that leptin acts as an endogenous “sensing” factor, linking the environment (availability of nutrients) to circulating Treg number. Since nutritional deprivation increases the susceptibility to infection and associates with the amelioration of clinical manifestations of inflammation and autoimmunity, it will be important to address how this condition relates to the influence of leptin on both Teff and Tregs. In MS, leptin secretion is increased in serum and CSF of naive-to-treatment subjects and this directly correlates with the secretion of IFN-γ in the CSF. Leptin levels inversely correlate with the percentage of circulating Treg cells, a subset able to dampen the autoimmune response mediated by myelin-specific Teff cells present in MS subjects, thus reinforcing the link between the number of Treg cells and leptin secretion *in vivo* (61). The findings that different intracellular metabolic pathways have an impact in the control of self-immune tolerance and the study of how metabolic dysregulation in overweight and obesity could alter immune tolerance are now topics of intensive investigation. Recent evidence suggests that metabolic and autoimmune diseases, characterized by chronic inflammation and altered self-immune tolerance, are more common in affluent countries; the reasons for such phenomena are still not completely understood, but the “metabolic disturbances” induced by nutritional overload, observed in more developed countries, seem to play the main role.

## Conclusion

Despite the overwhelming indirect evidence correlating MetS and inflammation to brain cognition, direct causative effects of metabolic imbalance in pathological alteration of the BBB remain to be established. Specifically, the metabolic pathways that affect the cellular component of the BBB, as well as those regulating T cell function and access to the CNS, are yet to be defined. The identification of molecules selectively altered by MetS will provide new targets for corrections/interventions in all those neurodegenerative disorders with a clear metabolic imbalance and autoimmune pro-inflammatory component as well as biomarkers of early-stage BBB malfunction with the aim of preventing disease onset and progression.

## Conflict of Interest Statement

The authors declare that the research was conducted in the absence of any commercial or financial relationships that could be construed as a potential conflict of interest.
